# Novel Humanized Recombinant T Cell Receptor Ligands Protect the Female Brain After Experimental Stroke

**DOI:** 10.1007/s12975-014-0345-y

**Published:** 2014-05-18

**Authors:** Jie Pan, Julie Palmateer, Timothy Schallert, Madison Hart, Arushi Pandya, Arthur A. Vandenbark, Halina Offner, Patricia D. Hurn

**Affiliations:** 1 Nanjing University Medical School, Nanjing, People’s Republic of China; 2 Department of Neuroscience, University of Texas at Austin, Austin, TX USA; 3 Department of Psychology, University of Texas at Austin, Austin, TX USA; 4 Department of Neurology, Oregon and Health Sciences University and Neuroimmunology Research, Portland, OR USA; 5 Department of Molecular Microbiology and Immunology, Oregon and Health Sciences University and Neuroimmunology Research, Portland, OR USA; 6 Department of Anesthesiology and Perioperative Medicine, Portland Veterans Affairs Medical Center, Oregon and Health Sciences University and Neuroimmunology Research, Portland, OR USA; 7 University of Texas System, Austin, TX USA; 8 Research and Innovation University of Texas System, 601 Colorado, Suite 211, Austin, TX 78701 USA

**Keywords:** Cerebral ischemia, Gender, Sex, Immunotherapy, Partial MHC class II constructs, Stroke, Ultrasonic vocalization

## Abstract

Transmigration of peripheral leukocytes to the brain is a major contributor to cerebral ischemic cell death mechanisms. Humanized partial major histocompatibility complex class II constructs (pMHC), covalently linked to myelin peptides, are effective for treating experimental stroke in males, but new evidence suggests that some inflammatory cell death mechanisms after brain injury are sex-specific. We here demonstrate that treatment with pMHC constructs also improves outcomes in *female* mice with middle cerebral artery occlusion (MCAO). HLA-DR2 transgenic female mice with MCAO were treated with RTL1000 (HLA-DR2 moiety linked to human MOG-35-55 peptide), HLA-DRa1-MOG-35-55, or vehicle (VEH) at 3, 24, 48, and 72 h after reperfusion and were recovered for 96 h or 2 weeks post-injury for measurement of histology (TTC staining) or behavioral testing. RTL1000- and DRa1-MOG-treated mice had profoundly reduced infarct volumes as compared to the VEH group, although higher doses of DRa1-MOG were needed for females vs. males evaluated previously. RTL1000-treated females also exhibited strongly improved functional recovery in a standard cylinder test. In novel studies of post-ischemic ultrasonic vocalization (USV), as measured by animal calls to their cage mates, we modeled in mice the post-stroke speech deficits common in human stroke survivors. The number of calls was reduced in injured animals relative to pre-MCAO baseline regardless of RTL1000 treatment status. However, call duration was significantly improved by RTL1000 treatment, suggesting benefit to the animal’s recovery of vocalization capability. We conclude that both the parent RTL1000 molecule and the novel non-polymorphic DRα1-MOG-35-55 construct were highly effective immunotherapies for treatment of transient cerebral ischemia in females.

## Introduction

Inflammation is not only an important mechanism that contributes to brain damage after cerebral ischemia but also an important element in protection and repair. It is now well established that significant brain injury, including ischemic stroke in humans and animals [1–3], triggers rapid activation of the peripheral immune system, resulting in migration of immunocytes such as T and B lymphocytes, monocytes, and neutrophils into the damaged area [4–6]. Furthermore, post-ischemic CNS activation of the peripheral immune system is followed by immunosuppression that is marked by drastic atrophy of lymphoid tissue such as spleen and thymus in animals [3, 7] and is under investigation in humans [8]. Thus, immunotherapy that targets the inflammatory milieu of the brain and which averts derangement of immune function would be highly promising with potential applications to the clinic.

Partial major histocompatibility complex class II (pMHC) constructs, including recombinant T cell receptor ligands (RTL) molecules, have been designed with α1 and β1 domains of the MHC class II molecules expressed as a single polypeptide with (or without) antigenic peptide extensions [9, 10]. These constructs have been well studied in experimental autoimmune encephalomyelitis (EAE), in phase 1 clinical trials for multiple sclerosis (MS) [11, 12], and currently are in phase 2 efficacy trials for MS. We reported previously that RTL551 (pI-A^b^/mMOG-35-55) administered 4 h after middle cerebral artery occlusion (MCAO) improved experimental injury outcomes [13, 14]. More importantly for human stroke, RTL1000 (an HLA-DR2 moiety linked to human MOG-35-55 peptide) reduced infarct size and improved behavioral outcomes in humanized DR2 mice [15]. We have recently shown that RTL1000 binds to and downregulates CD74, the natural receptor for macrophage migration inhibitory factor, MIF, on CD11b + cells, thus strongly inhibiting MIF-dependent inflammation [16, 17].

One impediment to the use of these recombinant proteins in humans is the requirement to match the recipient patient’s MHC class II with the polymorphic β1 domain of the RTL construct in order to reduce possible alloreactivity. To circumvent this restriction, a novel RTL was developed that contains the non-polymorphic HLA-DRα1 but not the HLA-DR2β1 domain, thus preventing any possible alloreactivity to β1, linked to the MOG-35-55 peptide [18]. This construct reduced infarction after transient MCAO in male mice [19].

All of these studies have been carried out exclusively in male animals. It is now well known that some molecular mechanisms of cell death after brain injury are sex-specific and that not all therapies work equally in male vs. female animals [for reviews see 20–22]. Recently, we observed that there are significant differences in the female vs. male immune response to ischemic brain injury. Specifically, male rodents inhibit larger infarction after MCAO [23, 24] and, perhaps consequently, are more vulnerable than females to post-ischemic spleen destruction and early, large transmigration of monocytes (macrophages and dendritic cells) from spleen to brain. Some of these processes differ in the female (6). Accordingly, the purpose of the present study was to evaluate therapeutically relevant pMHC in female mice and determine efficacy in reducing histological damage and behavioral deficits. Such studies are required if these agents are to be employed in the clinic in patients of both sexes.

## Methods

Female DR2 mice were randomized to receive 100 μg RTL1000 or TRIS-buffer vehicle (VEH 0.1 ml) by subcutaneous injection at 3, 24, 48, and 72 h of reperfusion post-MCAO. Animals for the HLA-DRα1-MOG-35-55 study were randomized to receive 100 μg, 500 μg, 1 mg, or vehicle at 3, 24, 48, and 72 h of reperfusion. Drug doses and treatment intervals were based on previous studies in males.

Transient focal cerebral ischemia was induced via 60 min of reversible right MCAO using the intraluminal filament technique (6–0 suture) under isoflurane anesthesia as previously described [15]. Temporalis muscle temperature was maintained at 35.5–37.5 °C throughout MCAO with a heating lamp, and occlusion/reperfusion was confirmed by laser-doppler flowmetry (LDF). Sham-operated mice were handled in the same way as MCAO-treated animals with the exception of cauterizing the common, external, and internal arteries. During the recovery period, all mice were evaluated for general health status on days 1 to 3 after surgery, as previously described [15].

### Histology

Animals were survived for 96 h post-MCAO and then euthanized under deep isoflurane anesthesia for 2,3,5-triphenyltetrazolium chloride (TTC) histology, as previously described [15]. Both sides of each slice were photographed with a digital camera. Infarction volume was determined by digital photography and image analysis (Image J, NIH, USA) and integrated across four slices. The mice were excluded if intracranial or subarachnoid hemorrhage was found. Infarct volume was expressed as a percentage of the contralateral structure.

### Behavioral Studies

Mice undergoing behavioral testing were single housed in a 12/12-h light–dark cycle, and all assessments were carried out by an observer blinded to treatment during the second half of the light cycle (1200 to 1800 h). The corner turn test was used to determine unilateral sensorimotor dysfunction, as previously described with some modifications [25]. Briefly, the animal is encouraged into a narrow-angled corner, forcing the animal to turn to its uninjured side. Each animal was tested by ten trials timed for pre-MCAO, day 8 post-MCAO, and day 15 post-MCAO. To supplement the corner turn test but allow for greater sensitivity for early post-MCAO sensory motor deficits, we developed a novel tube test that determines an animal’s preference for sidedness when facing a dead end. The animal was placed in a horizontal tube with one end closed off by a Plexiglas square. The tube itself is 20 cm long with a diameter of 5 cm; narrow enough to encourage the animal to reach the tube end but large enough for turning. An uninjured animal will turn to left or right with equal likelihood, while an animal post-MCAO will turn to the contralateral side as it experiences decreased sensory and motor control in the injured side. Animals were tested pre-MCAO and at days 1, 2, 3, and 8 post-MCAO. The pre-test consisted of ten trials; after each complete turn, the animal was removed from the tube for 30 s of rest before the next trial. Testing at days 1, 2, and 3 post-MCAO consisted of only five trials due to decreased mobility, while testing at day 8 returned to a full ten trials.

The cylinder test was used as previously described [26] to analyze forelimb use bias pre-MCAO and at days 3, 7, and 14 post-MCAO. Briefly, the animal was placed in a transparent cylinder that allows rearing behavior. Using video-recording, paw touches to the cylinder are counted as the animal rears and supports its weight. A count of “both” was added when the animal used both paws simultaneously or needed to add the second paw to help support itself. Each test was concluded when 20 touches were counted or at 10 min. Final scores were calculated as right turns as a percentage of the animal’s pre-MCAO score.

To test sensory-cognitive function, the social novel odor recognition task was used as previously described [27]. Briefly, wooden balls were placed in the animal’s cage to absorb the “home” odor. In contrast, novel odor balls were curated from an alternative cage housing mice not known to the animal. On day 1 of testing, each animal was individually placed in the home cage with four home odor balls for three 1-min trials. One novel odor ball was then introduced to the animal on days 5 and 6 post-MCAO. Trials were again conducted with the novel odor, allowing the animal to habituate to this odor. Twenty-four hours later, the animal was again exposed to a home odor ball and the previously novel odor ball. Time spent exploring each of the balls was recorded and analyzed.

### Recording Mouse Ultrasonic Vocalization (USV)

Animals were tested in a dark recording chamber which consists of a 29 × 18 × 25 cm^3^ acrylic cage divided into upper and lower subchambers with a microphone poised in the lower subchamber. USVs were monitored and recorded using software (Avisoft-SAS Lab Pro, Berlin Germany). Mice were housed in groups of four, as in other behavior tests. For testing, a mouse is separated 1 day prior to recording and then placed in the lower subchamber while stimulated by its smell and calls of its previous cage mates in the upper subchamber over 50 mins of recording time. Mice were tested 1 day pre-MCAO as a baseline, followed by repeat testing at 3 days post-MCAO. Only mice with robust vocalization (greater than 50 “calls” in the 50-min recording session) were included in the testing. The total number of calls, call duration, and frequency bandwidth (frequency range 30–120 kHz) were analyzed for each session, then compared between sham-treated, vehicle-treated, or RTL1000-treated MCAO mice.

### Statistical Analysis

Data are presented as the mean ± SEM. Histological and physiological data were analyzed with two-tailed Student’s *T* test for the two treatment groups (e.g., infarction) and analysis of variance (ANOVA) corrected for multiple comparisons by post hoc Tukey’s test for all groups (e.g., LDF, temporalis muscle temperature). Behavior testing was analyzed by two-way repeated measures ANOVA with a post hoc Student Newman Keuls test to correct for multiple comparisons. All statistical analyses were carried out using IBM SPSS Statistics (version 21.0; IBM, New York, NY, USA). The criterion for statistical significance was *p* ≤ 0.05.

## Results

1. RTL1000 strongly reduces infarction (Fig. 1): RTL1000 reduced infarct volumes (expressed as percent of contralateral structure): in total hemisphere 14.3 ± 4.1 % in RTL (*n* = 15) vs. 26.8 ± 2.9 % in VEH (*n* = 15) (*P* = 0.009). Reduced infarction was apparent in cortex (19.9 ± 6.3 % in RTL vs. 44.2 ± 5.0 % in VEH (*P* = 0.005)) and striatum (24.2 ± 6.8 % in RTL vs. 52.4 ± 4.7 % in VEH group (*P* = 0.002)). Mice with no measurable infarction evident at 96 h numbered 6/15 in the RTL group, but only 1/15 in the vehicle-treated group. There were no differences in intraischemic LDF (15 ± 1.0 % in RTL vs. in 13 ± 1.3 % VEH (*P* > 0.05)) or temporalis muscle temperature (37.0 ± 0.2 in RTL vs. 36.7 ± 0.2 in VEH (*P* > 0.05)). No difference was found in weight and health scores between groups. Mortality was 1 in RTL and 1 in VEH. Two mice were excluded due to LDF ≥ 35 % during the surgery process, and six were excluded due to subarachnoid hemorrhage (four in RTL group and two in VEH group).

**Fig. 1:**
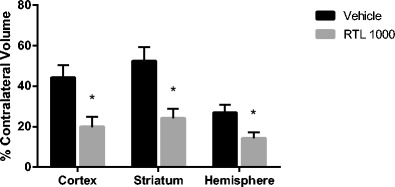
Effects of RTL1000 on infarct volumes. Mice were subjected to 60 min-MCAO and followed by four daily treatments of vehicle (Tris–HCl) (*n* = 15) or 100 μg RTL1000 (*n* = 15). The infarct volume was quantified as a percentage of the non-ischemic contralateral cortex, striatum, or hemisphere, respectively. Values are mean ± SEM. *P ≤ 0.05
2. HLA-DRα1-MOG-35-55 reduces infarction in a dose-dependent manner (Fig. 2): 1 mg treatment with HLA-DRα1-MOG-35-55 reduced infarct volume relative to vehicle treatment, in total hemisphere (22.8 ± 4.4 % in HLA-DRα1-MOG-35-55 (*n* = 10) vs. 33.1 ± 1.3 % in VEH (*n* = 14) (*P* = 0.02)); in cortex (34.9 ± 7.3 % vs. 56.5 ± 2.9 % (*P* = 0.007)); and in striatum (48.2 ± 8.0 % vs. 69.7 ± 3.7 % (*p* = 0.02)). In contrast, 100 μg (*n* = 12) and 500 μg (*n* = 11) doses improved infarction relative to the VEH group. No difference was observed in intraischemic LDF among the various groups (12 ± 0.7 % in 100 μg vs. 11 ± 0.6 % in 500 μg vs. 9 ± 0.4 % in 1 mg vs. 11 ± 0.6 % in VEH (*P* > 0.05)) and temporalis muscle temperature (36.1 ± 0.2C° in 100 μg vs. 36.0 ± 0.2C° in 500 μg vs. 36.2 ± 0.1C° in 1 mg vs. 36.2 ± 0.1C° in VEH (*P* = 0.05)). No differences in body weight, health scores, or mortality rates were observed among the groups. The only deaths occurred in the vehicle-treated and 500 μg treated group (two in each group).

**Fig. 2:**
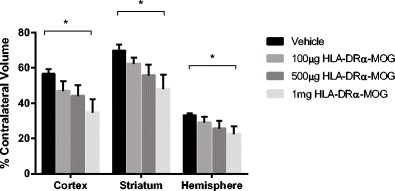
Dose-dependent effects of HLA-DRα1-MOG-35-55 on infarct volumes. Mice were subjected to 60 min-MCAO and followed by four daily treatments of vehicle (Tris–HCl) (*n* = 14), 100 μg (*n* = 12), 500 μg (*n* = 11), and 1 mg (*n* = 10) HLA-DRα1-MOG-35-55. The infarct volume was quantified as a percentage of the non-ischemic contralateral cortex, striatum, or hemisphere, respectively. Values are mean ± SEM. *P ≤ 0.05
3. Neurobehavioral testing was conducted on the following 16 day survival cohorts: MCAO RTL1000 = 16, MCAO vehicle = 17, sham RTL1000 = 10, and sham vehicle = 10. In both the corner turn and tube turn tests, injured animals show a preference for turning towards the injured side as the animal uses its uninjured limbs to turn its body (Table 1). MCAO animals in both treatment groups showed this preference at 8 days post-stroke (MCAO vehicle 75.9 ± 6, sham vehicle 43 ± 6.3, MCAO RTL1000 77.5 ± 6, sham RTL1000 56 ± 4; *P* = 0.05), but the effect was ameliorated at day 15 post-stroke. In the tube turn test (Fig. 3), both the MCAO treatment groups showed a preference to turn to the injured side as compared to their treatment-matched shams.

**Table 1:** Percentage of right turns in tube turn and corner turn tests. The tube turn test showed an effect of stroke at day 2 in both MCAO groups as compared to their matched shams. The effect of stroke was ameliorated over time for the MCAO RTL1000-treated mice (*n* = 16), while MCAO VEH-treated mice (*n* = 17) still showed increased right preference 8 days after stroke

	Tube turn					Corner turn		
	Pre-stroke	Post-stroke day 1	Post-stroke day 2	Post-stroke day 3	Post-stroke day 8	Pre-stroke	Post-stroke day 8	Post-stroke day 15
Sham vehicle	53 (±9.6)	56 (±7.8)	44 (±9.8)	48 (±10.8)	38 (±7.7)	58 (±4.7)	43 (±6.3)	51 (±5.7)
Sham RTL1000	44 (±8.2)	62 (±9.2)	49.7 (±4.4)	68 (±5.3)	60 (±6)	46 (±4.5)	56 (±4)	56 (±6)
MCAO vehicle	44.1 (±6.4)	68.2 (±8.6)	70.6 (±8.6)*	69.4 (±8.1)	64.7 (±7)*	46.5 (±5.6)	75.9 (±6)*	61.2 (±6.1)
MCAO RTL1000	55.2 (±4.9)	72.5 (±8.9)	76.3 (±7.4)*	75 (±8.1)	64.4 (±6.6)	50.6 (±3.4)	77.5 (±6)*	61.9 (±5.9)

The corner turn test showed an effect of stroke 8 days after MCAO which was ameliorated at 15 days after MCAO in both groups. *P ≤ 0.05 (Sham VEH (*n* = 10); sham RTL (*n* = 10)) Values are mean ± SEM

**Fig. 3:**
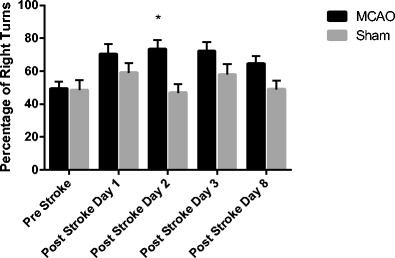
Tube turn test, MCAO vs. sham. Mice were tested 1 day before MCAO (pre), 24 h after MCAO (post-stroke day 1), 48 h after MCAO (post-stroke day 2), 72 h after MCAO (post-stroke day 3), and 8 days after MCAO (post-stroke day 8). MCAO-treated mice (*n* = 33) show a significantly increased preference for right turns at day 2 (*P = 0.001) compared to sham-operated mice (*n* = 20). Values are mean ± SEM
4. The cylinder test was conducted prior to surgery and then on days 3, 7, and 14 post-operatively. There were no differences in the pre-test scores (not shown) or between sham groups at any time point (Fig. 4). The vehicle-treated MCAO group showed preference for the right (uninjured) forelimb at every post-operative time point, as expected (*P* values = 0.05). The RTL1000-treated MCAO group showed no difference in forelimb preference as compared to sham at any time point but was significantly different from the vehicle-treated MCAO group at days 3, 7, and 14 (*P* = 0.05). The improvement in RTL-treated MCAO group as compared to the VEH group grew larger over the course of testing.

**Fig. 4:**
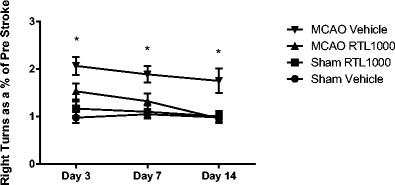
Cylinder test. MCAO-vehicle-treated mice (*n* = 17) show significantly increased right forelimb use compared to sham-vehicle-treated mice (*n* = 10) at all time points, while MCAO-RTL-treated mice (*n* = 16) show no significant difference from the sham-RTL-treated mice (*n* = 10). Results shown as a ratio to pre-MCAO test scores, i.e., post-stroke day 3/pre. Testing took place 1 day before MCAO (pre), 3, 7, and 14 days after MCAO. *P < 0.05 compared to matched sham. Values are mean ± SEM
5. Considering the specificity of RTL1000’s effects and the unilateral nature of the injury, a drug effect was not expected in the novel odor recognition test (Table 2). While all four groups were more interested in the novel odor than the familiar odor during the first trial, both the MCAO groups had less exploration of the novel odor than their matched sham groups (MCAO vehicle 4.8 ± 1.4, sham vehicle 9.7 ± 1.5, MCAO RTL1000 3.5 ± 0.9, sham RTL1000 7.3 ± 2.5 (*P* = 0.05)). By the third trial, all groups of animals had habituated to the novel odor, showing no time difference spent on the novel vs. familiar odors. Twenty-four hours after the first exposure to the novel object, there were no differences in time spent exploring novel or home odors between any groups (Fig. 5).

**Table 2:** Novel odor recognition after MCAO. At the introduction of the novel odor (day 1–1) MCAO VEH (*n* = 17) and MCAO RTL (*n* = 16) both show less interest in the novel odor than their respective shams (*P ≤ 0.05)

	HO	Day 1–1	Day 1–3	Day 2
Sham vehicle	1.1 (±0.1)	9.7 (±1.5)	1.7 (±0.4)	2 (±0.6)
Sham RTL1000	0.8 (±0.1)	7.3 (±2.5)	1.9 (±0.5)	1.8 (±0.4)
MCAO vehicle	0.7 (±0.1)	4.8 (±1.4) *	0.6 (±0.1)	1.2 (±0.3)
MCAO RTL1000	0.4 (±0.1)	3.5 (±0.9) *	1.3 (±0.5)	1.3 (±0.4)

There are no other significant differences between groups among home odor (HO), day 1 trial 3 of the novel odor (day 1–3), or novel odor after 24 h latency (day 2). (Sham VEH (*n* = 10), sham RTL (*n* = 10), MCAO VEH (*n* = 17), MCAO RTL (*n* = 16)). Values are mean ± SEM

**Fig. 5:**
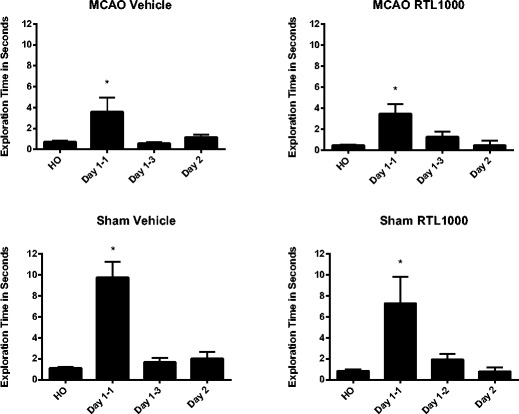
Novel odor recognition after MCAO. Exploration time of odor balls shown for each treatment group. Mice were initially exposed to the novel odor 5 days after stroke and then tested for scent memory retention 24 h later, 6 days after MCAO. All groups spent more time on the novel odor compared to their home odor when first exposed; however, this interest diminished by the third trial on day 1 of exposure as the animal habituated to the odor. Twenty-four hours later, all groups showed this habituated response to the same novel odor. *HO* = home odor, the animal’s own scent. *Day 1–1* = novel odor on day 1, 1st trial. *Day 1–2* = novel odor on day 1, 3rd trial. *Day 2* = novel odor 24 h latency. *P ≤ 0.05 (sham VEH (*n* = 10), sham RTL (*n* = 10), MCAO VEH (*n* = 17), MCAO RTL (*n* = 16)). Values are mean ± SEM
6. USV was recorded in each animal at baseline and at day 3 post-MCAO or sham-MCAO and then analyzed for the number of “calls,” individual call duration, and bandwidth of calls observed over the recording session. Baseline call number varied from 50 to 1,640 (MCAO RTL1000 314 ± 110, MCAO vehicle 341 ± 189, sham 101 ± 18). By day 3, call number was greatly reduced in post-MCAO animals, regardless of RTL treatment status (Fig. 6). Similarly, day 3 call duration was reduced in injured animals, although this effect was improved by RTL1000 treatment (*P* = 0.05). At day 3, the bandwidth of recorded calls ranged widely, and there was no difference between groups.

**Fig. 6:**
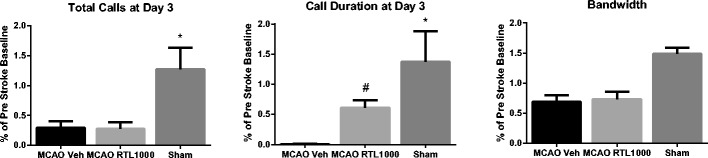
Ultrasonic vocalizations before and after MCAO. Measurements at post-MCAO day 3 of total calls, call duration, and bandwidth are shown as a percentage of the baseline (pre-surgery recording). *Asterisk* indicates that both MCAO groups demonstrate lower number of calls (MCAO vehicle vs. sham (*P* = 0.00); MCAO RTL1000 vs. sham (*P* = 0.00)) and shorter duration of calls as compared to sham-operated mice. (MCAO vehicle vs. sham (*p* = 0.00); MCAO RTL1000 vs. sham (*p* = 0.03)) RTL treatment, but not vehicle treatment, partially restores call duration to that of sham-operated mice. *Number sign* indicates MCAO RTL1000 different from MCAO VEH (*p* = 0.05) (sham (*n* = 5), MCAO VEH (*n* = 8)) MCAO RTL1000 (*n* = 8). Values are means ± SEM

## Discussion

The results presented above demonstrate several important findings. First, both the RTL1000 and DRα1-MOG-35-55 constructs reduced infarct volume in female mice with a therapeutic window of 3 h post-MCAO. This is consistent with our previous reports of efficacy in male mice [15, 19]. Second, we show for the first time in either sex that RTL1000 strongly improves behavioral outcome, as assessed by the cylinder test. The improvement in behavior is specific to motor function, as would be expected by the unilateral striatal/cortical injury that typically is produced by unilateral MCAO.

Third, we studied post-ischemic USV, as measured by animal calls to their cage mates, as a novel method to model in animals the post-stroke speech deficits so common in humans. The number of calls was reduced in injured animals relative to pre-MCAO baseline, regardless of RTL treatment status. However, call duration was improved by RTL treatment, suggesting a beneficial effect of RTL on the animal’s recovery of robust vocalization capability. We conclude that both the parent RTL1000 molecule and the novel DRα1-MOG-35-55 constructs were highly effective immunotherapies for treatment of transient cerebral ischemia in females.

These results are of particular importance given recently identified differences in female vs. male immune responses to ischemic brain injury that include less-prominent inflammatory responses and smaller infarct volumes in females. However, the data clearly demonstrate that such immunological differences in infarct development did not alter the efficacy of RTL1000 that involves inhibition of transmigration of monocytes and macrophages from spleen to brain during MCAO. Thus, this study strongly supports the clinical application of RTL1000 and possibly DRa1-MOG-35-55 to stroke patients of both sexes.

Although treatment of MCAO using four daily injections of 100 μg RTL1000 produced highly significant reductions in infarct volumes in both females (current study) and males [15], successful treatment of MCAO with the DRα1 construct given daily for 4 days required a higher dose in females (1 mg) than in males (100 μg). The reasons for this difference remain unclear but could be related to gender differences in inflammation that are mediated through MIF/CD74 interactions or MIF-independent mechanisms. We have recently shown that both RTL1000 and DRα1-MOG-35-55 bind to and downregulate CD74, the natural receptor for macrophage migration inhibitory factor, MIF, on CD11b + monocytes, macrophages, and activated microglial cells. This binding removes available CD74 from the cell surface, directly inhibits MIF binding to CD74, and blocks downstream MIF effects in the CNS during EAE [17, 18]. However, it is not yet clear to what extent, if any, the treatment effect of RTL1000 and DRα1-MOG-35-55 on MCAO in females and males is mediated through MIF blockade.

**Fig. 7:**
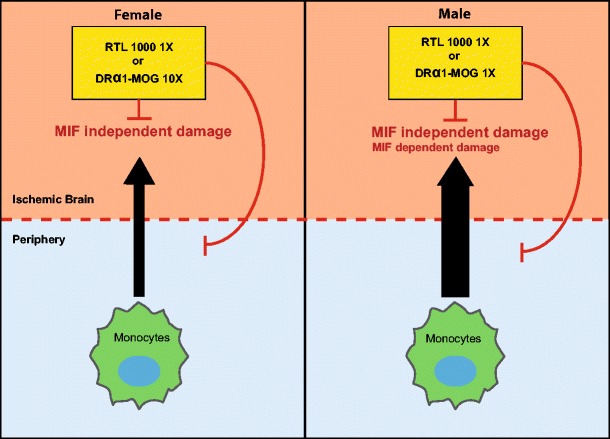
RTL1000 and DRα1-MOG-35-55 treatment of MCAO. RTL1000, which is comprised of the HLA-DR2 β1α1 domains linked to human MOG-35-55 peptide and DRα1-MOG-35-55 that lacks the polymorphic β1 domain, can reduce infarct size in female and male mice after MCAO. In males, where peripheral immune cells cause greater MCAO damage than in females and possibly some MIF dependent inflammation, RTL1000 and DRα1-MOG-35-55 have the same treatment potency. In females, which appear to have little if any MIF-dependent MCAO damage, treatment with RTL1000 is tenfold more potent than DRα1-MOG-35-55. This differential treatment effect between males and females could be based on gender-associated effects of MIF on infarct size, recruitment of peripheral immune cells into the ischemic brain, and axonal death

Indeed, the total picture of MIF involvement in MCAO, although still incomplete, appears to include both deleterious effects during the first week after MCAO not directly linked to inflammatory mechanisms (reduced infarct size in male MIF-KO mice 7 days but not 3 days after MCAO, direct intraneuronal MIF activity after oxygen glucose deprivation leading to neuronal cell death, [28, 29] and protective effects (worse stroke outcome in female MIF-KO mice, [30])). These findings suggest that MIF may only be involved in the acute early phase of stroke in males, with effects mainly on neurons, whereas MIF would not appear to play any pathogenic role in females with MCAO (Fig. 7). In this light, it is apparent that the strong treatment effect of RTL1000 and DRα1-MOG-35-55 in MCAO is largely MIF independent, especially in female mice.

In males, there may be some possible MIF-dependent effects that might include protection from neuronal damage as we have observed in the EAE, optic neuritis, and macular degeneration models [31–33]. Although it is unclear what are the MIF-independent pathways in MCAO affected by RTL1000 and DRα1 constructs, conceivably they could involve inhibition of MOG-specific inflammatory T cells through the tethered MOG-35-55 peptide or increased random migration of macrophages induced by reduction of CD74 expression similar to that observed in macrophages from CD74 KO mice [34].

We utilized sensory, motor, and memory testing, as well as a novel assessment of vocalization in the female mice. The cylinder test has been well used as a means of evaluating motor function in injured rodents, and the data clearly show a large beneficial effect of RTL1000 on functional recovery. RTL1000-treated mice fully recovered relative to their sham counterparts, unlike vehicle-treated animals. As expected, tests which centered on early recovery (tube and corner turning) demonstrated a pervasive effect of MCAO in all animals, emphasizing that the mechanism(s) behind RTL1000’s efficacy are not due to overall animal health or morbidity.

Similarly, the RTL1000-treated mice did not perform better in a test of sensory memory, as measured by the novel odor recognition test, emphasizing that RTL1000 only improved functional outcomes in areas specific to MCAO-inflicted damage.

Our studies are the first to characterize the effect of cerebral ischemia on the USV that ordinarily occurs in a sex-specific and stimulus-specific manner in healthy rats and mice [35, 36]. In the female mouse, baseline vocalization to cage mates is readily measurable, although there is large variance in this behavior among animals. Accordingly, we normalized each animal’s data obtained at post-MCAO day 3 to baseline. MCAO clearly suppressed both the number and duration of calls in all animals, and RTL1000 mitigated this abnormality in part. In contrast, vocalization was not reduced in sham-operated animals, and in some shams, calling to cage mates relative to baseline. These abnormalities may be specific to experimental stroke, as we have previously observed in male rats treated with unilateral haloperidol-induced dopamine depletion or unilateral 6-hydroxydopamine injection that dopaminergic dysfunction narrowed call bandwidth without reducing the number of calls [37]. Male rats treated with caffeine or amphetamine demonstrate altered 50-kHz vocalization or “trill calls” [38, 39]. Normal USV in mice has been connected to the expression of the FOXP2 gene, encoding a transcription factor containing a polyglutamine tract and fork head DNA-binding domain [36, 40]. Further study is required to evaluate the mechanism of abnormal post-ischemic USV or if altered FoxP2 expression plays a role in these abnormalities.

In summary, both the parent RTL1000 molecule and the novel DRα1-MOG-35-55 constructs were highly effective immunotherapies for treatment of MCAO in females, in spite of the requirement for a tenfold higher dose of the DRα1-MOG-35-55 construct to achieve comparable effects. Moreover, the non-polymorphic DRα1-MOG-35-55 construct lacking the polymorphic DR2 β1 domain would be better suited for treatment of stroke patients without the need for HLA screening prior to use, an impediment that would limit use of RTL1000 to pre-screened DR2+ patients.

## References

[1] Iadecola C, Anrather J (2012). The immunology of stroke: from mechanisms to translation. Nat Med.

[2] Macrez R, Ali C, Toutirais O, Le Mauff B, Defer G, Dirnagl U (2011). Stroke and the immune system: from pathophysiology to new therapeutic strategies. Lancet Neurol.

[3] Offner H, Vandenbark AA, Hurn PD (2009). Effect of experimental stroke on peripheral immunity: CNS ischemia induces profound immunosuppression. Neuroscience.

[4] Ren X, Akiyoshi K, Dziennis S, Vandenbark AA, Herson PS, Hurn PD (2011). Regulatory B cells limit CNS inflammation and neurologic deficits in murine experimental stroke. J Neurosci.

[5] Ren X, Akiyoshi K, Grafe MR, Vandenbark AA, Hurn PD, Herson PS (2011). Myelin specific cells infiltrate MCAO lesions and exacerbate stroke severity. Metab Brain Dis.

[6] Banerjee A, Wang J, Bodhankar S, Vandenbark AA, Murphy SJ, Offner H (2013). Phenotypic changes in immune cell subsets reflect increased infarct volume in male vs female mice. Transl Stroke Res.

[7] Offner H, Subramanian S, Parker SM, Afentoulis ME, Vandenbark AA, Hurn PD (2006). Experimental stroke induces massive, rapid activation of the peripheral immune system. J Cereb Blood Flow Metab.

[8] Sahota P, Vahidy F, Nguyen C, Bui TT, Yang B, Parsha K (2013). Changes in spleen in patients with acute ischemic stroke: a pilot observational study. Int J Stroke.

[9] Burrows GG, Adlard KL, Bebo BF, Chang JW, Tenditnyy K, Vandenbark AA (2000). Regulation of encephalitogenic T cells with recombinant TCR ligand. J Immunol.

[10] Burrows GG, Chou YK, Wang C, Chang JW, Finn TP, Culbertson NE (2001). Rudimentary TCR signaling triggers default IL-10 secretion by human Th1 cells. J Immunol.

[11] Yadav V, Bourdette DN, Bowen JD, Lynch SG, Mattson D, Preiningerova J (2012). Recombinant T-cell receptor ligand (RTL) for treatment of multiple sclerosis: a double blind, placebo-controlled, phase 1, dose-escalation study. Autoimmun Dis.

[12] Offner H, Sinha S, Burrows GG, Ferro AJ, Vandenbark AA (2011). RTL therapy for multiple sclerosis: a phase 1 clinical study. J Neuroimmunol.

[13] Subramanian S, Zhang B, Kosaka Y, Burrows G, Grafe M, Vandenbark A (2009). Recombinant T cell receptor ligand treats experimental stroke. Stroke.

[14] Dziennis S, Mader S, Akiyoshi K, Ren X, Ayala P, Burrows GG (2011). Therapy with recombinant T-cell receptor ligand reduces infarct size and infiltrating inflammatory cells in brain after middle cerebral artery occlusion in mice. Metab Brain Dis.

[15] Akiyoshi K, Dziennis S, Palmateer J, Ren X, Vandenbark A, Offner H (2011). Recombinant T cell receptor ligands improve outcome after experimental cerebral ischemia. Transl Stroke Res.

[16] Vandenbark AA, Meza-Romero R, Benedek G, Andrew S, Huan J, Chou YK (2013). A novel regulatory pathway for autoimmune disease: binding of partial MHC class II constructs to monocytes reduces CD74 expression and induces both specific and bystander T-cell tolerance. J Autoimmun.

[17] Benedek G, Meza-Romero R, Andrew S, Leng L, Burrows GG, Bourdette D (2013). Partial MHC class II constructs inhibit MIF/CD74 binding and downstream effects. Eur J Immunol.

[18] Meza-Romero R, Benedek G, Yu X, Mooney J, Dahan R, Duvshani N, Bucala R, Offner H, Reiter Y, Burrows GG, Vandenbark AA. HLA-DRa1 constructs block CD74 expression and MIF effects in experimental autoimmune encephalomyelitis. J Immunol. 2014;192(9):4164–73. 10.4049/jimmunol.1303118 PMC4028955 24683185

[19] Benedek G, Zhu W, Libal N, Casper A, Yu X, Meza-Romero R, Vandenbark AA, Alkayed NJ, Offner H. A novel HLA-DRα1-MOG-35-55 construct treats experimental stroke. Met Brain Dis 2014;29:37–45. 10.1007/s11011-013-9440-0 PMC3975671 24122483

[20] Herson PS, Palmateer J, Hurn PD (2013). Biological sex mechanisms of ischemic brain injury. Transl Stroke Res.

[21] Cheng J, Hurn PD (2010). Sex shapes experimental ischemic brain injury. Steroids.

[22] Manwani B, Liu F, Scranton V, Hammond MD, Sansing LH, McCullough LD (2013). Differential effects of aging and sex on stroke induced inflammation across the lifespan. Exp Neurol.

[23] Alkayed NJ, Harukuni I, Kimes AS, London ED, Traystman RJ, Hurn PD (1998). Gender-linked brain injury in experimental stroke. Stroke.

[24] McCullough LD, Zeng Z, Blizzard KK, Debchoudhury I, Hurn PD (2005). Ischemic NO and PARP activation in cerebral ischemia: male toxicity, female protection. J Cereb Blood Flow Metab.

[25] Zhang L, Schallert T, Zhang ZG, Jiang Q, Arniego P, Li Q (2002). A test for detecting long-term sensorimotor dysfunction in the mouse after focal ischemia. J Neurosci Methods.

[26] Schallert T, Fleming SM, Leasure JL, Tillerson JL, Bland ST (2000). CNS plasticity and assessment of forelimb sensorimotor outcome in unilateral rat models of stroke, cortical ablation, parkinsonism and spinal cord injury. Neuropharmacology.

[27] Spinetta MJ, Woodlee MT, Feinberg LM, Stroud C, Schallert K, McCormick LK (2008). Alcohol induced retrograde memory impairment in rats: prevention by caffeine. Psychopharmacology.

[28] Inacio AR, Bucala R, Deierborg T (2011). Lack of macrophage migration inhibitory factor in mice does not affect hallmarks of the inflammatory/immune response during the first week after stroke. J Neuroinflammation.

[29] Inacio AR, Ruscher K, Leng L, Bucala R, Deierborg T (2011). Macrophage migration inhibitory factor promotes cell death and aggravates neurologic deficits after experimental stroke. J Cereb Blood Flow Metab.

[30] Turtzo LC, Li J, Persky R, Benashski S, Weston G, Bucala R (2013). Deletion of macrophage migration inhibitory factor worsens stroke outcome in female mice. Neurobiol Dis.

[31] Wang C, Gold BG, Kaler LJ, Yu X, Afentoulis ME, Burrows GG (2006). Antigen-specific therapy promotes repair of myelin and axonal damage in established EAE. J Neurochem.

[32] Adamus G, Brown L, Andrew S, Meza-Romero R, Burrows GG, Vandenbark AA (2012). Neuroprotective effects of recombinant T-cell receptor ligand in autoimmune optic neuritis in HLA-DR2 mice. Invest Ophthalmol Vis Sci.

[33] Adamus G, Wang S, Kyger M, Worley A, Lu B, Burrows GG (2012). Systemic immunotherapy delays photoreceptor cell loss and prevents vascular pathology in Royal College of Surgeions rats. Mol Vis.

[34] Fan H, Hall P, Santos LL, Gregory JL, Fingerle-Rowson G, Bucala R (2011). Macrophage migration inhibitory factor and CD74 regulate macrophage chemotactic responses via MAPK and Rho GTPase. J Immunol.

[35] Bowers JM, Perez-pouchoulen M, Edwards NS, McCarthy MM (2013). Fox P2 mediates sex differences in ultrasonic vocalization by rat pups and directs order of maternal retrieval. J Neurosci.

[36] Gaub S, Groszer M, Fisher SE, Ehret G (2010). The structure of innate vocalizations in Foxp2-deficient mouse pups. Genes Brain Behav.

[37] Ciucci MR, Ma ST, Fox C, Kane JR, Ramig LO, Schallert T (2007). Qualitative changes in ultrasonic vocalization in rats after unilateral dopamine depletion or haloperidol: a preliminary study. Behav Brain Res.

[38] Simola N, Ma ST, Schallert T (2010). Influence of acute caffeine on 50-kHZ ultrasonic vocalizations in male adult rats and relevance to caffeine-mediated psychopharmacological effects. Int J Neuropsychopharmacol.

[39] Ahrens AM, Ma ST, Duvauchelle CL, Schallert T (2009). Repeated intravenous amphetamine exposure: rapid and persistent sensitization of 50 kHz ultrasonic trills in rats. Behav Brain Res.

[40] Shu W, Cho JY, Jiang Y, Zhang M, Weisz D, Elder GA (2005). Altered ultrasonic vocalization in mice with a disruption in the Foxp2 gene. PNAS.

